# Characterization of a Novel Aspect of Tissue Scarring Following Experimental Spinal Cord Injury and the Implantation of Bioengineered Type-I Collagen Scaffolds in the Adult Rat: Involvement of Perineurial-like Cells?

**DOI:** 10.3390/ijms23063221

**Published:** 2022-03-16

**Authors:** Haktan Altinova, Pascal Achenbach, Moniek Palm, Istvan Katona, Emmanuel Hermans, Hans Clusmann, Joachim Weis, Gary Anthony Brook

**Affiliations:** 1 Institute of Neuropathology, RWTH Aachen University Hospital, 52074 Aachen, Germany; pascal.achenbach@rwth-aachen.de (P.A.); myri.palm@gmail.com (M.P.); ikatona@ukaachen.de (I.K.); jweis@ukaachen.de (J.W.); gbrook@ukaachen.de (G.A.B.); 2 Department of Neurosurgery, RWTH Aachen University Hospital, 52074 Aachen, Germany; hclusmann@ukaachen.de; 3 The Berlin Police, Medical Commission, 13589 Berlin, Germany; 4 Department of Neurology, RWTH Aachen University Hospital, 52074 Aachen, Germany; 5 Institute of Neuroscience, Université Catholique de Louvain, 1200 Brussels, Belgium; emmanuel.hermans@uclouvain.be

**Keywords:** spinal cord injury, microstructured collagen scaffold, CNS-scarring, perineurial-like cells, fibrotic encapsulation, implant interface

## Abstract

Numerous intervention strategies have been developed to promote functional tissue repair following experimental spinal cord injury (SCI), including the bridging of lesion-induced cystic cavities with bioengineered scaffolds. Integration between such implanted scaffolds and the lesioned host spinal cord is critical for supporting regenerative growth, but only moderate-to-low degrees of success have been reported. Light and electron microscopy were employed to better characterise the fibroadhesive scarring process taking place after implantation of a longitudinally microstructured type-I collagen scaffold into unilateral mid-cervical resection injuries of the adult rat spinal cord. At long survival times (10 weeks post-surgery), sheets of tightly packed cells (of uniform morphology) could be seen lining the inner surface of the repaired dura mater of lesion-only control animals, as well as forming a barrier along the implant–host interface of the scaffold-implanted animals. The highly uniform ultrastructural features of these scarring cells and their anatomical continuity with the local, reactive spinal nerve roots strongly suggest their identity to be perineurial-like cells. This novel aspect of the cellular composition of reactive spinal cord tissue highlights the increasingly complex nature of fibroadhesive scarring involved in traumatic injury, and particularly in response to the implantation of bioengineered collagen scaffolds.

## 1. Introduction

Despite extensive research, there is still no widely accepted clinical treatment to repair severe spinal cord injury (SCI). Animal models of SCI have revealed that dramatic changes at the lesion site include the immediate destruction of tissue followed by a sequence of secondary pathophysiological events including inflammation, oedema, cystic cavitation, and the development of molecular and physical barriers associated with glial and connective tissue scarring [1,2,3,4,5]. Loss of the natural orientated geometry of spinal cord white matter tracts (including the glial framework) at the lesion site and the formation of an axon growth-repulsive scarring environment have been reported to be major factors in the inhibition of axon regeneration and the lack of any functional tissue repair [6]. Characterization of the multiple and complex events taking place during secondary tissue degeneration has resulted in the development of numerous experimental strategies that have shown great promise in promoting tissue repair and functional recovery. Such strategies include blockade of myelin-associated axon growth-inhibitory molecules, enzymatic degradation of axon-repulsive extracellular matrix-related molecules (e.g., highly sulphated chondroitin sulphate proteoglycans, CSPGs), administration of axon growth-promoting factors, and implantation of stem cells and/or glia, as well as of engineered biomimetic scaffolds designed to replace critical aspects of anisotropic tissue geometry and bridge the lesion site [7]. All of these strategies result in the return of some degree of useful function in experimental animal models, and it is widely assumed that the most effective treatments will involve a combination of these approaches [8,9,10,11]. A major challenge in the field of tissue engineering and regenerative medicine is the ability of any implanted scaffold or device to interact appropriately with the surrounding host tissues, which critically depends on its competence in supporting intimate contact and integration with the key cellular elements of that tissue [12,13,14,15]. The present manuscript highlights a novel cellular aspect of the detrimental host scarring response to the implantation of a bioengineered scaffold in an experimental model of SCI.

Collagen has proven to be a popular natural polymer for bioengineering because of its abundance, its ability to be engineered into almost any three-dimensional form, and its inherent biological functionality and biodegradability [16]. Porcine collagen scaffolds composed of tightly packed, highly orientated micro-channels have been developed to bridge lesions of the peripheral nervous system (PNS) and central nervous system (CNS), and cytocompatibility has been tested in vitro with a range of neural cell types [13,14,15,17]. The implantation of porous, longitudinally microstructured collagen-based scaffolds as bridges across over-critical sized (i.e., 2 cm) nerve gaps of the adult rat sciatic nerve resulted in substantial axon regeneration and long-term functional tissue repair [12,18]. More recent clinical trials with collagen scaffolds have demonstrated its their [19,20]. In contrast, the ability of orientated microstructured collagen scaffolds to bridge experimental spinal cord injuries has been less successful, a significant complicating issue being the variable and limited integration between such implants and the surrounding host CNS tissues [21,22]. The interactions of scar-forming cells (particularly of the fibroadhesive cells) with implanted scaffolds is a subject of substantial importance, but one that remains poorly characterised and is a significant challenge in the development of biomimetic materials for traumatic SCI.

Earlier investigations of cellular reorganisation and scarring after SCI (i.e., in the absence of scaffold implantation) have demonstrated the development of dense astroglial and connective tissue scarring associated with the migration of a range of cell types into the lesion sites, including monocytes and microglial-derived macrophages, endothelial cells, ependymal cells, leptomeningeal fibroblasts, as well as Schwann cells originating from local, damaged spinal nerve roots [22,23,24,25,26,27]. More recently, genetic labelling techniques in animal models of SCI have indicated the additional involvement and important roles of type-A pericytes and perivascular fibroblasts, which become dissociated from microvessels and larger diameter blood vessels, respectively [28,29]. The involvement of any (or all) of these cells in the host scarring response to scaffold or device implantation is an issue of great importance that has only partially been characterised.

Although limited functional recovery has been observed over a period of 10 weeks following implantation of a longitudinally microstructured collagen scaffold in an in vivo model of SCI, this was likely due to some degree of tissue sparing and reduced cystic cavitation rather than any axon regeneration through the scaffold, since there was a lack of any substantial host neural tissue integration with the implant [30], a situation that could not be significantly improved by co-implanting purified populations of axon growth-promoting olfactory ensheathing cells [31]. Our more recent immunohistochemical studies of such long-term lesion/implantation sites have demonstrated sheets of fibroblast-like scarring cells of uncertain identity forming an intense zonula occludens-1 (ZO-1) immunoreactive transition zone between the collagen implant and the surrounding host tissues [32,33]. The general lack of suitable quality immunohistochemical markers for specific labelling of fibroblast sub-types has prompted us to test the hypothesis that a clearer understanding of the type and origin of the cells in the transition zone could be obtained by investigating their fine structural features by transmission electron microscopy (TEM). The aim of the study was to provide a more detailed characterization of the cells contributing to reduced implant–host integration, thereby broadening research opportunities to include the development of strategies capable of ameliorating such encapsulating or scarring responses.

The cells of the transition zone revealed an abundance of tight junctions between overlapping cells and their tightly packed processes, as well as numerous caveolae and a discontinuous or partial basal lamina sheath at the cell surface. Clusters and sheets of cells with such characteristics could be found lining the inner aspect of the reconstructed dura mater of lesion-only control animals, as well as extending for substantial distances along the implant–host interface of the implanted scaffold. The uniformity of these ultrastructural characteristics and the apparent anatomical continuity of these scarring cells with local, damaged spinal nerve roots strongly suggest their identity to be migrating perineurial-like cells (PNLC). This novel aspect of the cellular composition of lesioned spinal cord tissue highlights the complex nature of fibroadhesive scarring following traumatic SCI, and its unexpected role in blocking host tissue integration after implantation of biomimetic microstructured collagen scaffolds.

## 2. Results

**Figure 1:**
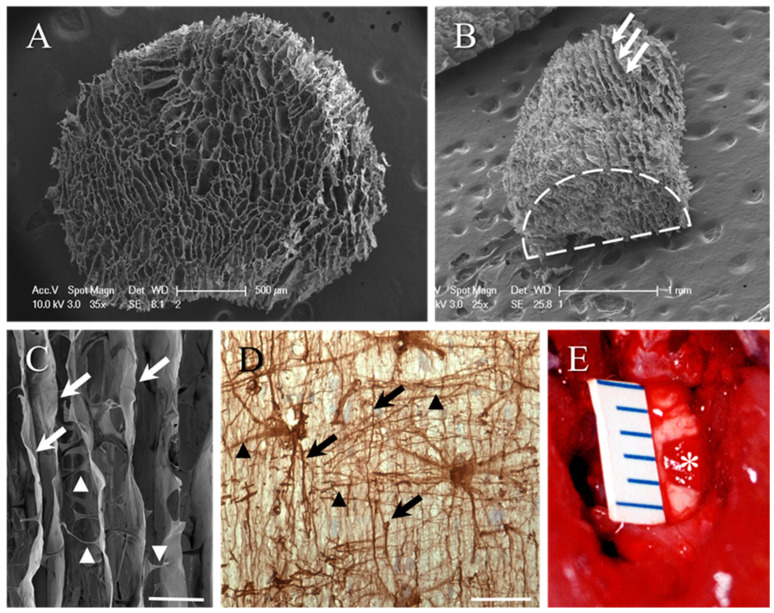
Biomimetic design of the microporous collagen scaffold. (**A**) SEM of the honeycomb-like appearance of the collagen scaffold cylinders after being removed from the 2 mm tissue punch (end-on view). (**B**) Tangential view of the scaffold hemi-cylinder generated by a mid-line incision along the axis of the cylinder. The profile of the end of the hemi-cylinder is highlighted by the dashed line. Longitudinal orientation of the porous framework can be identified on the scaffold surface (white arrows). (**C**) Higher-magnification SEM demonstrating the walls of longitudinally orientated scaffold framework (white arrows). The substantial fenestration between adjacent micropores is highlighted by the transversely orientated profiles (arrowheads). This bioengineered framework mimics, to some extent, the pattern and orientation of the astroglial framework of spinal cord white matter tracts as revealed by immunohistochemistry. (**D**) Glial fibrillary acidic protein (GFAP) immunohistochemistry of the interwoven pattern of longitudinal (black arrows) and transverse (black arrowheads) astrocytic processes in a longitudinal section of the lateral funiculus of the adult rat spinal cord. (**E**) A 2 mm long hemi-cylinder of the collagen scaffold (asterisk) fits neatly into the gap generated by the lateral funiculotomy of the adult rat cervical spinal cord, making excellent implant–host contact. The implant appears red after absorbing blood from the surrounding host tissue. The size of the lesion/implant is indicated by mm scale. Scale bars: **A** = 500 µm; **B** = 1 mm; **C** = 50 µm; **D** = 50 µm.

The microstructure of the collagen scaffold was visualised by scanning electron microscopy (SEM). The end-on view revealed the honeycomb-like appearance of the scaffold (Figure 1A) and tangential views demonstrated the aligned, longitudinal orientation of the microchannels (e.g., white arrows, Figure 1B). Higher-magnification SEM also revealed the significant fenestration of the microchannel walls, effectively linking adjacent channels (arrowheads, Figure 1C). This bioengineered framework mimics, to some extent, the pattern of the astroglial framework of unlesioned spinal cord white matter tracts, as revealed in longitudinal sections of the contralateral funiculus of the cervical spinal cord. Glial fibrillary acidic protein (GFAP) immunohistochemistry demonstrated the interwoven pattern of longitudinal (black arrows) and transversely directed (black arrowheads, Figure 1D) astrocytic processes within the white matter. A 2 mm long hemi-cylinder of the collagen scaffold (asterisk) could be implanted to fit neatly into the gap generated by the lateral funiculotomy of the adult rat cervical spinal cord, making excellent implant–host contact (Figure 1E).

**Figure 2:**
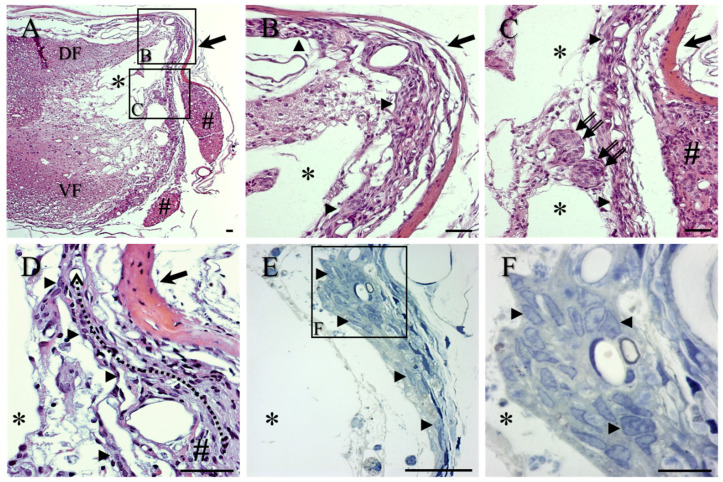
General morphology of lesion sites in lesion-only control animals, in transverse sections stained with H&E (**A**–**D**) and toluidine blue semi-thin sections (**E**,**F**). (**A**) Low-magnification image of fluid-filled cystic cavities which were often divided by trabeculae (asterisks). Local spinal nerve roots are indicated by # and eosinophilic repaired dura mater is indicated by large arrow. For orientation, the dorsal funiculus is indicated by DF and the ventral funiculus by VF. (**B**) The lateral edge of cystic cavities (asterisks) was lined by layers of cells (arrowheads) directly at the medial surface of the repaired eosinophilic dura mater (arrow). (**C**) The described cell layers were observed to be in continuity with local, damaged spinal nerve roots (#; see also (**A**) for general overview). Reactive clusters of cells, possibly representing mini-fascicles of regenerating nervous tissue, can also be seen (double arrows). (**D**) The meandering black arrow indicates the arrangement of the reactive cell layer (arrowheads) medial to the eosinophilic dura mater (arrow). (**E**) The nuclei of the cells within these layers were better visualised in the toluidine blue-stained semi-thin sections, and displayed a fine rim of dense heterochromatin surrounding a medium-pale euchromatin (e.g., arrowheads; shown at higher magnification in (**F**)). Scale bars: **A**–**E** = 50 µm; **F** = 20 µm.

The overall lesion site morphology of control animals was visualised by haematoxylin and eosin (H&E) staining of cryostat sections (Figure 2A–D) or by toluidine blue staining of semi-thin sections (Figure 2E,F). By 10 weeks post-operation (p.o.), fluid-filled cystic cavities, commonly containing trabeculae of scarring tissue, were regularly observed within the injury site (asterisks in Figure 2A–F). The lateral edge of the cystic cavities appeared to be lined by single or multiple layers of cells that were often juxtaposed to the inner aspect of the repaired dura mater (arrows, Figure 2A–D), passing around blood vessels (Figure 2B,E,F) and extending to dorso-lateral and ventro-lateral regions of the lesion site (e.g., arrowheads, Figure 2B–F). These layers were often observed to be in continuity with the reactive outer connective tissue layers of local, damaged spinal nerve roots (e.g., #, Figure 2A,C,D, meandering black arrow in D; see also Figure S1A,B in Supplementary Materials). The ovoid-shaped nuclei of the cells forming this layer displayed a fine rim of dense heterochromatin surrounding a medium-pale euchromatin (arrowheads, Figure 2F). Scattered along the trajectory of these layers of reactive cells were occasional rounded clusters of cells, possibly representing mini-fascicles of regenerating nervous tissue (double arrows, Figure 2C).

**Figure 3:**
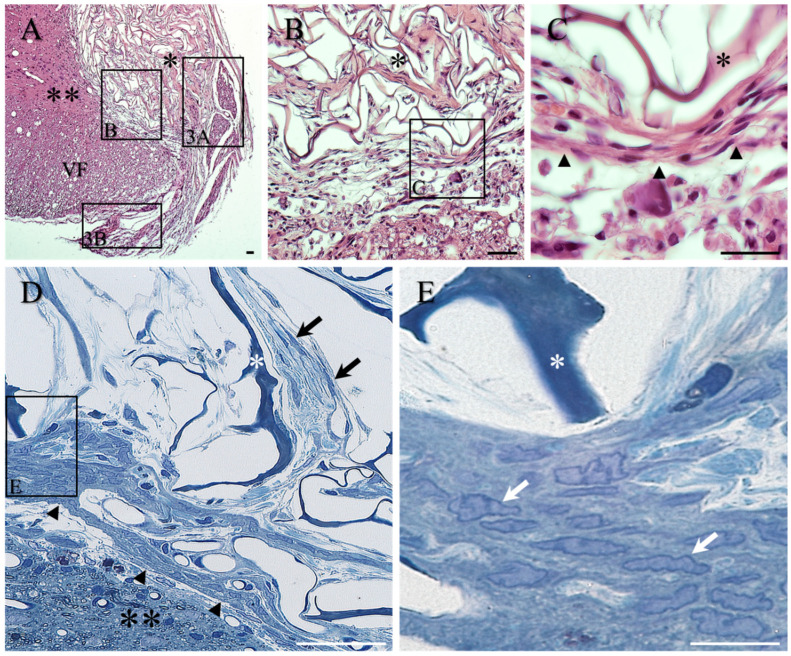
Morphology of implanted collagen scaffold in transverse sections stained with H&E (**A**–**C**) and for toluidine blue in semi-thin sections (**D**,**E**). (**A**) Low-magnification image of implanted scaffold (single asterisk) reveals the convoluted framework of the collagen scaffold and demonstrates the apparently good contact with the surrounding host spinal cord tissue (double asterisks). For orientation, the ventral funiculus is indicated by VF. Boxed area of the transition zone shown at higher magnification in Figure 2B. Other boxed areas shown at higher magnification in (**A**,**B**). (**B**) The presence of a conspicuous band of overlapping, elongated cells formed a transition zone at the interface between the collagen scaffold (asterisk) and the surrounding host tissue. Boxed area shown at higher magnification in (**C**). (**C**) Higher magnification of the band of the multiple overlapping cells (black arrowheads) coursing between the edge of the implant (asterisk) and the surrounding spinal cord tissue. (**D**) The morphology of the band of cells forming the transition zone around the implanted scaffold was more clearly seen in the toluidine blue semi-thin sections (black arrowheads). The darkly stained framework of the collagen scaffold (asterisk) highlights the open, porous nature of the scaffold with multiple, fine fibroblasts coursing amongst the palely stained collagen ECM deposits (black arrows). The overlapping cells and processes of the transition zone (arrowheads) separate the scaffold from the adjacent spinal cord parenchyma (double asterisk). (**E**) At high magnification, the uniform nuclear morphology of the tightly packed cells is apparent (white arrows). This observation is strikingly similar to the nuclear morphology of the cells in the lateral tissue bridge of the control group (compare with Figure 1F). Scale bars: **A**–**D** = 50 µm; **E** = 20 µm.

Both H&E and toluidine blue-stained sections revealed the generally good contact between the implanted scaffold and the surrounding host spinal cord tissue, as well as the convoluted microporous framework of the scaffold (single asterisks, Figure 3A–E). A common observation was the presence of a conspicuous band of overlapping, elongated cells that formed a transition zone along much of the interface between the implanted scaffold and the surrounding host tissue (Figure 3A–E; in particular, black arrowheads in Figure 3C,D).

This transition zone appeared to act as a cellular barrier, effectively separating the scaffold (indicated by a single asterisk) from the surrounding spinal cord parenchyma (indicated by double asterisks in Figure 3A,D). Within the porous framework of the scaffold, loosely scattered fibroblasts with elongated, fine processes could also be observed (e.g., black arrows, Figure 3D). High magnification of toluidine blue-stained semi-thin sections revealed the rather uniform, elongated nuclear appearance of the tightly packed cells of the transition zone, demonstrating a rim of dense heterochromatin surrounding a medium-dense euchromatin (e.g., white arrows, Figure 3E, being strikingly similar to the morphology of the cells in the lateral tissue bridge of the lesion-only control group; see Figure 2F).

**Figure 4:**
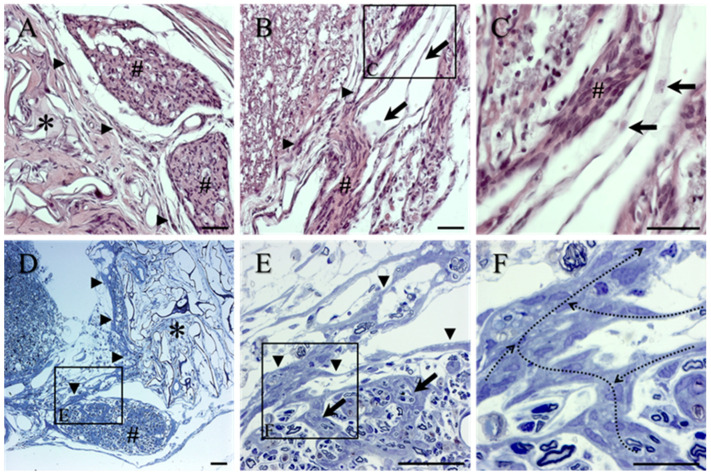
Morphology of reactive spinal nerve roots in transverse spinal cord sections from the collagen scaffold-implanted group stained with H&E (**A**–**C**) and for toluidine blue in semi-thin sections (**D**–**F**). (**A**) Dorsal spinal nerve roots (#) located between thin sheets of overlapping, elongated reactive cells (arrowheads) covering the outer-most surface of the implanted collagen scaffold (asterisk) and the repaired dura mater. (**B**) Pale staining of the leptomeninges (arrows, also seen at higher magnification in (**C**)). (**C**) Higher magnification of the leptomeninges (arrows) is demonstrated close to a damaged spinal nerve rootlet (#). Note the morphological differences between leptomeningeal cells and the reactive cells of the rootlet (#). (**D**) Areas of thickened, reactive perineurium that circumscribed part of a damaged ventral nerve root could be seen extending towards and along the medial edge of the implanted scaffold (arrowheads). The porous framework of the scaffold (asterisk) contains abundant deposits of lightly stained ECM. (**E**) Higher magnification of the boxed area in (**D**). The damaged nerve root is surrounded by the overlapping cells and processes of reactive PNLC (arrowheads), which also formed the so-called septae within the damaged, reactive roots (arrows). (**F**) Higher magnification of the boxed area in (**E**). The continuity of the reactive cells that formed the intra-spinal nerve root septae with the cells of the surrounding perineurium is indicated by the meandering dotted arrows. Scale bars: **A**–**E** = 50 µm; **F** = 20 µm.

Spinal nerve roots (indicated by # in Figure 4A–C for general H&E overview), located between the reconstructed dura mater and the reactive cells at the lateral-most edge of the collagen scaffolds (arrowheads, Figure 4A,B), were usually observed to be in continuity with the lateral tissue bridge. The pale H&E staining of the leptomeninges (e.g., arrows, Figure 4B, also in boxed area seen at higher magnification in Figure 4C) could be seen lying between a small, damaged spinal nerve rootlet (#, Figure 4B,C) and a more laterally positioned layer of reactive cells. The continuity of the cells of the transition zone with damaged spinal nerve roots was most clearly observed in the toluidine blue-stained sections of scaffold-implanted animals and provided an anatomical indication of the possible source of these scarring cells. Areas of thickened (reactive) perineurium that circumscribed part of a damaged nerve root could be seen extending towards and along the medial edge of the implanted collagen scaffold (arrowheads, Figure 4D,E). Closer inspection of the damaged nerve roots revealed the distribution of similar reactive, tightly packed cells that also formed septae, which coursed between and around mini-fascicles of regenerated axons (arrows, Figure 4E). By light microscopy, the cells of such intra-spinal nerve root septae also demonstrated the same nuclear staining pattern and tight packing as those of the reactive perineurium (arrowheads, Figure 4E; see also Supplementary Figure S2A) with which a direct line of anatomical continuity could be observed (e.g., black arrows, Figure 4E; continuity indicated as meandering dotted arrows, Figure 4F).

**Figure 5:**
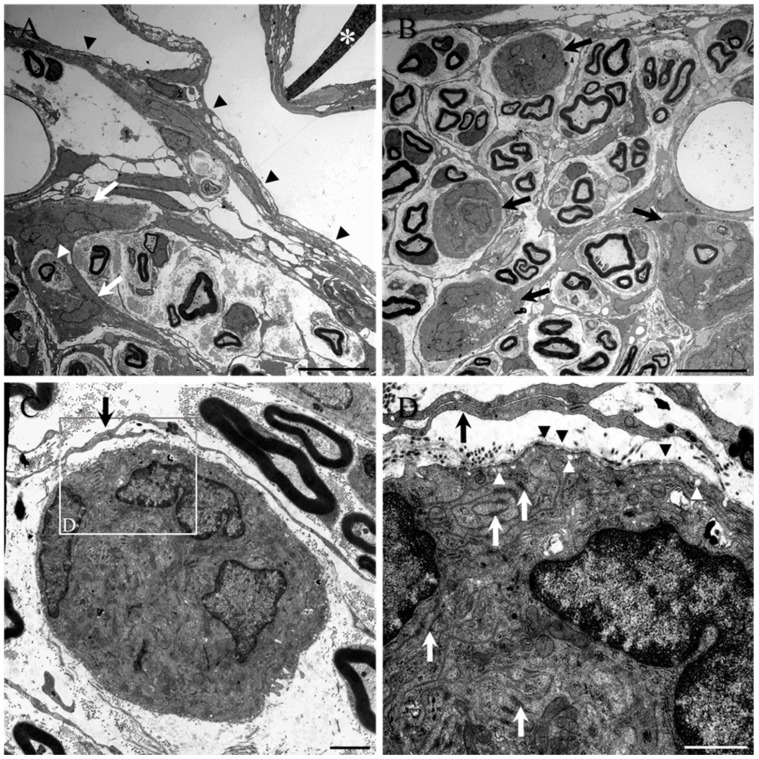
Ultrastructure of the inter-fascicular septae and cell clusters in damaged spinal nerve roots of the collagen scaffold-implanted group**.** (**A**) Transmission electron microscopy demonstrated the reactive perineurial septae (e.g., white arrows) of a damaged/regenerated spinal nerve root that is close to the implanted collagen scaffold (asterisk). The perineurial surface of the nerve root is indicated by black arrowheads. This particular septum can be seen partially surrounding a loosely packed group of Schwann cell-myelinated axons as well as a single, isolated axon (white arrowhead). (**B**) The reactive PNLC also form rounded clusters or nests of cells (black arrows) that can even form part of the regenerated mini-fascicles and are loosely encircled by the fine processes of fibroblast-like cells. (**C**) Higher magnification of a cell cluster containing 3 PNLC nuclei. (**D**) High magnification of boxed area in (**C**). Many electron-dense, tight junctions can be seen between the intricately interwoven and overlapping processes of the reactive cells (white arrows), and numerous pinocytic caveolae (white arrowheads) are also evident. A discontinuous basal lamina (black arrowheads) is also present over the surface of the cluster. These ultrastructural features were all strikingly similar to those of perineurial cells. The fine overlapping processes of the fibroblast-like cells that surround the mini-fascicle appear to lack a basal lamina and tight junctions. Scale bars: **A**,**B** = 10 µm; **C** = 2 µm; **D** = 1 µm.

Transmission electron microscopy revealed the morphology of the cells of the inter-fascicular septae within the damaged spinal nerve roots, as well as of the reactive perineurium (white arrows, Figure 5A and black arrowheads indicating the edge of the root; see also Supplementary Figure S2B) and also of rounded clusters or nests of cells (black arrows, Figure 5B). The reactive, closely packed cells of the septae, perineurium, and clusters possessed large nuclei with a dark rim of heterochromatin surrounding a medium-dense euchromatin. The cell clusters could be seen to partially or completely surround collagen fibril-rich mini-fascicles, individual myelinated axons, and blood vessels. Some of the clusters even appeared as part of the mini-fascicle complex, being encircled by fine fibroblast-like processes which lacked a basal lamina (Figure 5B; see also black arrow in Figure 5C,D). Mid-power and higher-power TEM of the reactive cell clusters demonstrated their tight packing of interwoven processes and dense, organelle-rich cytoplasm (Figure 5C and boxed area seen at higher magnification in Figure 5D). Many electron dense tight junctions between the convoluted and overlapping processes of the cells (white arrows, Figure 5D), numerous pinocytic vesicles or caveolae (sometimes associated with the outermost plasma membrane of the clustered cells, white arrowheads, Figure 5D) and the presence of a discontinuous or partial basal lamina (black arrowheads, Figure 5D, see also Supplementary Figure S2C,D) were routinely observed.

**Figure 6:**
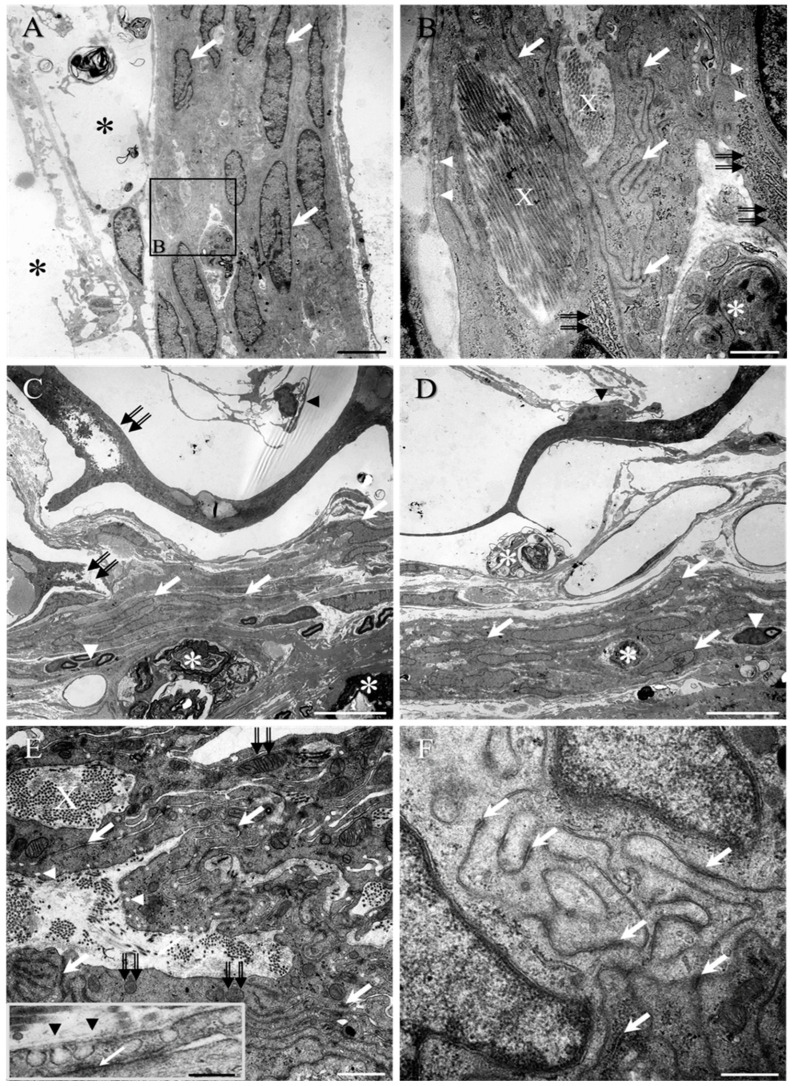
Ultrastructure of PNLC at the medial surface of the dura mater from the lesion-only control group (**A**,**B**) and at the transition zone from the collagen scaffold-implanted group (**C**–**F**). (**A**) Multiple overlapping reactive PNLC forming the tissue bridge at the medial (or inner) edge of the repaired dura mater of lesion-only control animals. Cystic cavitation is indicated by asterisks. The similar directionality of the ovoid-shaped nuclei (white arrows) suggests that the cells had all adopted the same orientation. (**B**) Higher magnification of boxed area in (**A**). Bundles of collagen fibrils (X; see also within the transition zone of (**E**)) appear trapped within lacunae located between the cells, as do phagocytic macrophages (asterisk). Large numbers of electron-dense, tight junctions (white arrows; see also (**E**,**F**) for transition zone) are formed between the fine overlapping cell processes, and many pinocytotic vesicles can be seen close to the plasma membrane (white arrowheads; see also (**E**) for the transition zone). An abundance of rER (double black arrows) is also present within the cell body. (**C**,**D**) The same PNCL-like features, with predominantly ovoid-shaped nuclei, were observed in the transition zone around the implanted scaffolds (white arrows). The collagen framework of the scaffolds showed signs of degradation (e.g., double black arrows in (**C**)), with fibroblast-like cells and their long, fine processes being located within the lumen of the porous framework or adherent to the collagenous walls (black arrowheads in (**C**,**D**), respectively). Small groups of Schwann cell-myelinated axons (white arrowheads in (**C**,**D**), respectively) and phagocytic macrophages (asterisks in (**C**,**D**)) were also embedded amongst the overlapping cells and processes. (**E**,**F**) Pockets or lacunae of dense and loosely packed collagen fibrils (X) were trapped between the cell processes, which were connected by multiple electron-dense, tight junctions (white arrows in (**E**) and insert in (**F**)). Numerous pinocytotic vesicles (white arrowheads in (**E**); see also insert) and mitochondria (double arrows in (**E**)) suggested high levels of transport and metabolic activity. A discontinuous basal lamina was also regularly observed (e.g., black arrowheads, insert in (**E**)). Scale bars: **A** = 5 µm; **B** = 1 µm; **C**,**D** = 10 µm; **E** = 1 µm (insert, 200 nm); **F** = 500 nm.

**Figure 7:**
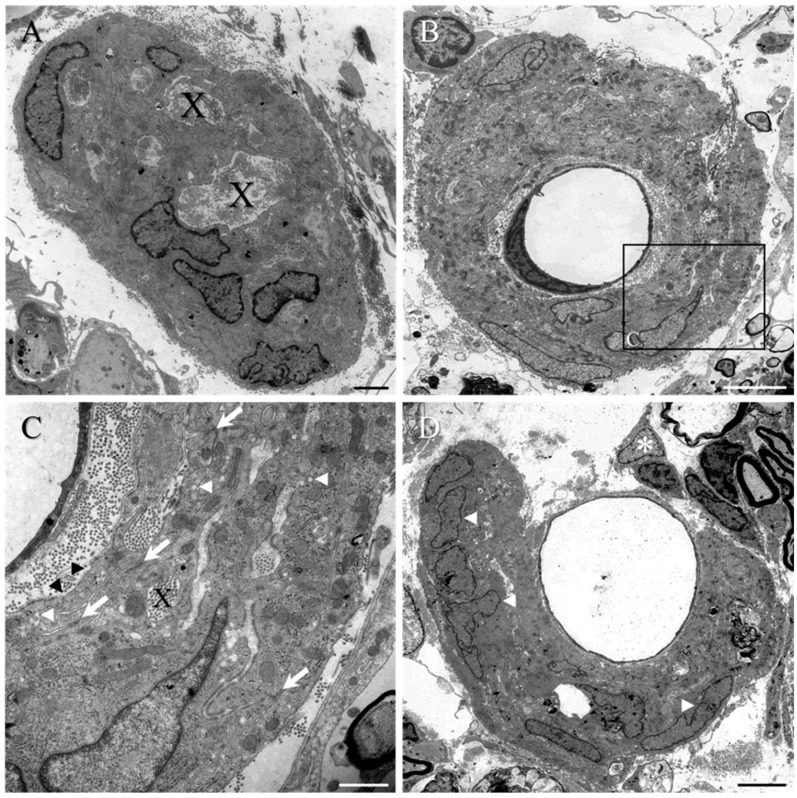
Ultrastructure of PNLC clusters or nests close to the implant of the collagen scaffold-implanted group. (**A**) As typically seen with this population of reactive cells, isolated clusters or nests of reactive PNLC commonly included bundles of collagen fibrils within lacunae (X in (**A**,**C**)). (**B**) Some clusters or nests of PNLC were also observed to totally envelop capillaries within the damaged spinal cord parenchyma. (**C**) High magnification of boxed area in (**B**). Lacunae of trapped collagen (X) were scattered amongst the numerous overlapping processes with tight junctions (white arrows), pinocytic vesicles (white arrowheads), and discontinuous basal lamina (black arrowheads). (**D**) Some clusters only partially enveloped capillary walls. However, the nuclei of the PNLC (white arrowheads) appeared to be distinct when compared to that of a pericyte (asterisk) that had divested itself from the vessel wall. Scale bars: **A** = 2 µm; **B**,**D** = 5 µm; **C** = 1 µm.

The ultrastructural features of the cells of the intra-nerve root septae, cell clusters, and reactive perineurium were all strikingly similar and consistent with those of perineurial cells. These PNLC features were also consistently observed in the cells along the inner lining of the dura mater of lesion-only control animals (Figure 6A,B), as well as of the transition zone (Figure 6C–F) located around the implanted scaffold, which showed signs of degradation (double black arrows, Figure 6C). In both regions, the nuclei of these reactive cell layers were predominantly ovoid in shape, all following the same orientation (white arrows, Figure 6A,C,D). The PNLC features were also present in the cells of isolated clusters, close to the transition zone or even associated with blood vessels (Figure 7A–D).

**Figure 8:**
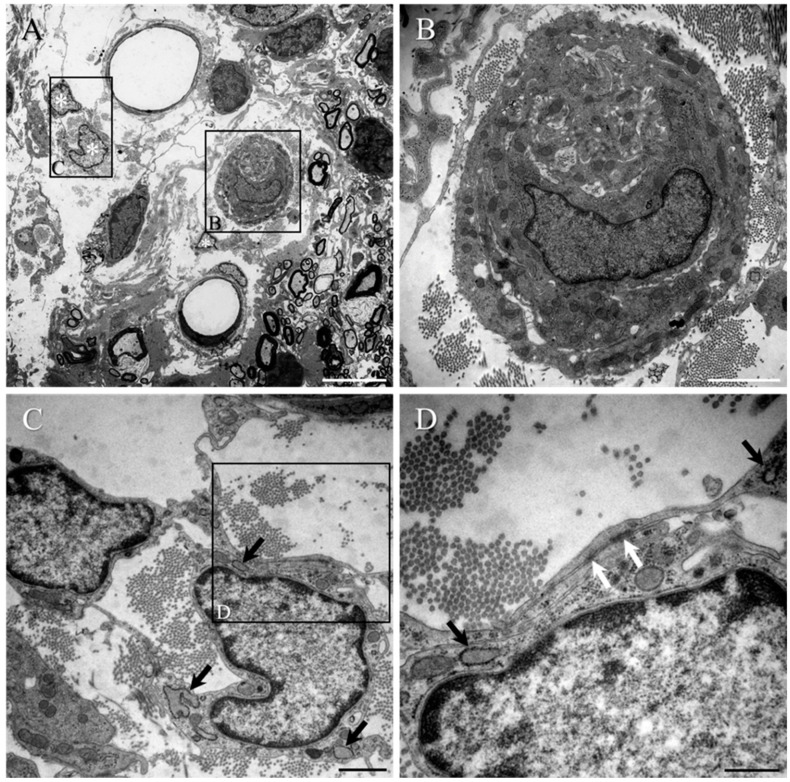
(**A**) A small PNLC cluster and a pair of reactive pericytes can be seen within the heavily disorganised white matter that was located close to the transition zone of the collagen scaffold-implanted group. (**B**) High magnification of boxed area in (**A**). The cell cluster is surrounded by areas of densely packed collagen fibrils. The typical morphological appearance of the single PNLC nucleus (containing medium-dense euchromatin) is clearly different from that of the pericytes (containing much paler euchromatin) that have dissociated themselves from the local microcirculation (compare the nucleus in (**B**) with those in (**C**)). (**C**) High magnification of boxed area in (**A**). The cell body of the reactive pericyte contains relatively little cytoplasm but numerous free ribosomes and rER with conspicuously dilated cisternae (black arrows; see also (**D**)). (**D**) High magnification of boxed area in (**C**). The fine overlapping processes of the reactive pericytes displayed occasional electron-dense, tight junctions (white arrow). Scale bars: **A** = 5 µm; **B** = 2 µm; **C** = 1 µm; **D** = 500 nm.

A number of structures were regularly encountered that appeared to be trapped within the layers or clusters of reactive PNLC, including bundles of collagen fibrils within lacunae (indicated as X in Figure 6B,E or in Figure 7A,C), phagocytic macrophages (asterisks, Figure 6B–D), as well as individual and small groups of Schwann cell-myelinated axons (white arrowhead, Figure 6C,D). The presence of abundant free ribosomes, rough endoplasmic reticulum (rER) (double black arrows, Figure 6B), glycogen granules, and numerous mitochondria (e.g., double black arrows, Figure 6E; see also Supplementary Figure S3) suggested that the PNLC were highly metabolically active, possibly in the building and maintenance of the large number of tight junctions (white arrows, Figure 6B,E,F or Figure 7C), the caveolae (white arrowheads, Figure 6B,E; see also insert in Figure 6E or Figure 7C), and the production of basal lamina (e.g., insert in Figure 6E, or Figure 7C). Images of the blood vessel-associated PNLC clusters also revealed the morphology of other local, reactive cell populations, such as pericytes (e.g., asterisk, Figure 7D, Figure 8) and astrocytes (Supplementary Figure S4A–D), both of which were somewhat morphologically different to the PNLC.

**Figure 9:**
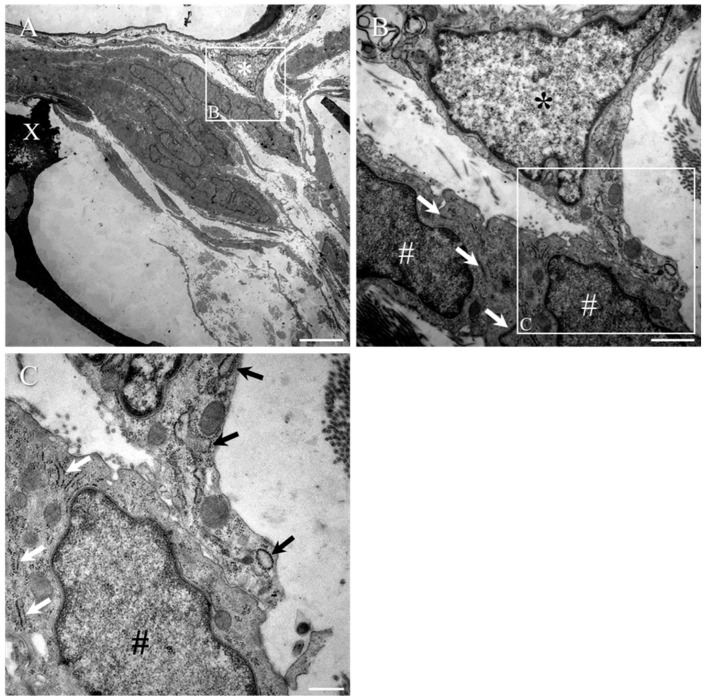
Reactive pericytes at the transition zone of the collagen scaffold-implanted group. (**A**) A reactive pericyte (asterisk) can be seen in close contact to a capillary vessel wall that was located adjacent to the PNLC of the transition zone around the partially degraded collagen scaffold (X). (**B**) High magnification of boxed area in (**A**). The moderately electron-dense nuclei of the PNLC (indicated by #) were readily distinguishable from the paler, electron-lucent nucleus of the pericyte (asterisk), which could be seen extending towards and making contact with the PNLC. Electron-dense, tight junctions between the PNLC were detectable (white arrows), even at moderately low magnification. (**C**) High magnification of the boxed area in (**B**). The pericyte process, extending from the cell body, displayed the characteristically dilated rER (arrows) that was at least double the width of that of the PNLC. The process of the pericyte made close contact with the plasma membrane of PNLC, but no tight junctions could be observed. Scale bars: **A** = 5 µm; **B** = 1 µm; **C** = 500 nm.

The small, reactive PNLC cluster seen in Figure 8A (shown at higher magnification in Figure 8B) is situated close to oligodendrocyte-myelinated axons of disorganised white matter, in which several reactive pericytes had become dissociated from local blood vessel walls (white asterisks, Figure 8A, shown at higher magnification in Figure 8C,D). Such pericytes displayed fine processes, and little cell body cytoplasm that contained moderate numbers of mitochondria (Figure 8C,D), but was notable due to the presence of a substantially dilated rER (black arrows, Figure 8C,D). The fine pericyte processes extended for short distances and contacted other cells, such as nearby PNLC (see Figure 9A–C) or other reactive pericytes (Figure 8C). No clear indications of tight junction formation between reactive pericytes and PNLC could be found (Figure 9B and at higher magnification in Figure 9C). However, in stark contrast, contact between the processes of adjacent reactive pericytes (Figure 8C, seen at higher magnification and white arrows in Figure 8D) were associated with tight junction formation. In other areas of reactive spinal tissue, examples of multiple layers of reactive pial cell processes (black arrows, Supplementary Figure S5A, seen at higher magnification in Supplementary Figure S5B) could also be observed next to the glia limitans (black arrowheads, Supplementary Figure S5A). Interestingly, the rER of these cells also appeared to be dilated (black arrows, Supplementary Figure S5B, but also compare this with the normal-looking rER of the PNLC, white arrows, Supplementary Figure S3), and numerous tight junctions could also be observed between their overlapping processes (white arrows, Supplementary Figure S5B).

Figure 8 shows a reactive pericyte (asterisk, Figure 9A) in close proximity to the wall of a capillary lying adjacent to the transition zone, around the partially degraded collagen scaffold (X, Figure 9A). The tight junctions between adjacent PNLC (#, in Figure 9B,C) processes of the transition zone are readily identifiable (white arrows, Figure 9B); however, such structures cannot be found where the processes of pericytes made close contact with PNLC (boxed area in Figure 9B, seen at higher magnification in Figure 8C). As mentioned earlier, the rER of reactive pericytes appeared to be markedly dilated (black arrows, Figure 9C), whereas that of the PNLC appeared substantially thinner (white arrows, Figure 9C).

## 3. Discussion

Severe traumatic SCI disrupts major populations of long descending and ascending nerve fibres as well as the orientated glial framework of white matter tracts, causing an acute loss of motor, sensory, and autonomic function. The subsequent formation of reactive tissue scarring and cystic cavitation results in the development of molecular and physical barriers to axonal regeneration and long-term neurological deficits [34,35,36,37]. It is widely acknowledged that future therapeutic interventions are likely to involve a combination of strategies [9,38], including the use of bioengineered scaffolds to assist in the bridging of the lesion site [11,39]. A major challenge in the development and design of implantable scaffolds or devices is ensuring that they can interact appropriately and efficiently with the surrounding host neural tissues, an issue that critically depends on their ability to form intimate contact and integration. The present manuscript highlights a novel aspect of the detrimental host cellular scarring response to the implantation of a bioengineered collagen scaffold that effectively separates the implant from the surrounding host neural tissues in an experimental model of spinal cord injury. It is anticipated that this will provide opportunities to the scientific, medical, and engineering communities to develop new areas of research in which the cellular and molecular mechanisms involved in such behaviour can be better understood and characterized as novel targets in future reparative intervention strategies.

The choice of implant used in the present investigation was driven by promising observations in the development and application of directionally microstructured collagen scaffolds to support the repair of traumatically injured PNS. Implantation of the scaffold into critical-sized defects of the rat PNS have supported significant morphological and functional repair in experimental animal models [12,30,40,41], and the scaffolds have been demonstrated to be tolerated and safe when implanted in clinical trials [19]. The longitudinally orientated scaffold micro-channels were generated by a patented method of unidirectional ice crystal formation [42] and mimicked the connective tissue architecture of peripheral nerves [14]. Interestingly, the longitudinal and transversely orientated framework of the highly porous and interconnected micro-channels mimic, to some extent, the “interwoven” astroglial framework of CNS white matter tracts [43], and its visco-elastic properties have been reported to be similar to those of mammalian spinal cord [17,44]. The high degree of fenestration between adjacent, longitudinally orientated micro-channels not only supported directed cell migration deep inside the scaffold, but also allowed the diffusion of sufficient nutrients to maintain the viability and proliferation of migrating cells. This has been indicated by extensive in vitro studies that have demonstrated collagen scaffold cytocompatibility with a range of rodent and human neural cell types, including Schwann cells, olfactory nerve ensheathing cells (ONECs), and astrocytes, as well as the scaffold’s ability to support directed axonal regeneration by regenerating motor and sensory axons [15,17,45,46].

Although a number of laboratories have focused on the implantation of orientated, micro-porous collagen scaffolds in an attempt to bridge complete or partial experimental spinal cord lesions, only a limited degree of functional recovery has been observed, and even this could not be correlated with axonal regeneration through the implants [21,22,47,48,49]. Leptomeningeal fibroblasts were assumed to be responsible for preventing axonal re-growth [21,22], and even our own morphometric immunohistochemical studies supported the fibroblast-like nature of these scarring cells [32,33]. By 10 weeks after scaffold implantation into the adult rat cervical spinal cord, only limited penetration by host neuronal and glial elements could be demonstrated. The ingrowth of blood vessels into the implant, however, achieved a density that was similar to that of unlesioned, contralateral white matter tracts, i.e., a level of vascularisation that was appropriate for the tissue that had been replaced by the implant [32]. Although host vascular integration was clearly supported by the scaffold, the presence of an intensely ZO-1 immunoreactive layer (or transition zone) of fibroadhesive scar tissue at the implant interface appeared to remain the major barrier to implant–host neural tissue integration [3]. These studies highlight the importance of research that is focussed on the interface between the host tissues and the implanted device.

The unilateral spinal cord resection model employed in this study not only caused a substantial defect of the lateral cervical funiculus, but also damaged local dorsal and ventral spinal nerve roots. This was apparent in H&E and toluidine blue-stained sections, as well as in TEM of the ultra-thin sections. Damaged spinal nerve roots demonstrated alterations such as the formation of tightly packed cell clusters that formed septae between and around regenerated mini-fascicles. Damaged nerve roots also typically displayed thickened, reactive perineurial sheaths that extended along the medial aspect of the repaired dura mater and penetrated for short distances as small, rounded cell clusters/nests into nearby areas of heavily damaged CNS. Such tightly packed layers of cells were particularly prominent as they extended from the damaged spinal nerve roots and formed an interface or transition zone that effectively separated the implanted scaffold from the adjacent, damaged spinal tissues. These observations support our earlier investigations that demonstrated a layer of tightly packed fibroblast-like scarring cells forming at the host–implant interface that were vimentin-positive, GFAP-negative, S100-negative, and intensely ZO-1-immunoreactive [32,33]. The anatomical distribution and relatively uniform ultrastructural features of such scarring cells (see below) suggest that they are derived from the reactive perineurium.

Tissue changes similar to those of the present study have been described in cases of perineurioma and intraneural perineurioma, benign peripheral nerve sheath tumours that are restricted to perineurial cells [50,51]. In such cases, perineurial cells often arranged themselves into pseudo-onion bulb-like whorls around Schwann cells and axons in varying stages of degeneration [51]. Furthermore, collagen-containing cells have been identified as perineurial cells in a case of atypical Cogan’s syndrome, sometimes associated with endoneurial blood vessels connected to the surrounding perineurium by perineurial septae [52]. Electron microscopy of the tightly packed cells, which formed the reactive septae within damaged spinal nerve roots, and of the thickened perineurium surrounding these nerve roots in the present study revealed a uniform morphology, with cell nuclei containing medium-dense euchromatin surrounded by a rim of heterochromatin, abundant, tight junctions between numerous thin, overlapping cell processes, multiple pinocytotic vesicles, and a discontinuous basal lamina, representing some of the typical ultrastructural characteristics of perineurial cells [53,54,55,56,57]. These same ultrastructural characteristics were also demonstrated by the sheets of tightly packed cells that formed the transition zone between the implanted collagen scaffold and the adjacent spinal cord parenchyma, as well as by the band of cells that formed along the medial aspect of the repaired dura mater of lesion-only control animals. Although electron microscopy has, since the mid-2000s, been considered the gold standard technique for confirming the identity of perineurial cells in pathological conditions such as perineurioma and its variants [51], our preferred terminology for the reactive, scarring-type cells observed in the present study is perineurial-like cells (PNLC).

The involvement of PNLC in the fibrotic scarring response suggests substantial migration of these cells from the locally damaged spinal nerve roots as a novel component of the cascade of secondary degenerative events following experimental SCI, but being particularly prominent following the implantation of the bioengineered collagen scaffold. Although there is little documentation about their migratory behaviour, perineurial cells have been reported to respond rapidly to experimental PNS resection injuries. Along with inflammatory cells, perineurial cells are amongst the earliest cell populations to migrate into implanted hollow silicone conduits that were used to bridge lesioned proximal and distal sciatic nerve stumps [55,58]. Early perineurial cell migration was subsequently followed by endothelial cells, Schwann cells, and regenerating axons. A similarly rapid migration of perineurial fibroblasts into the gap between severed, non-repaired peripheral nerve stumps has been described, in which ephrin-B/EphB signalling mechanisms have been shown to influence migrating Schwann cells. Such perineurial cell to Schwann cell signalling was reported to be pivotal for the formation of columns of Schwann cells and processes that could guide regenerating axons across the gap [59]. The ability of PNS-related glia to migrate into the injured spinal cord has been demonstrated in postmortem human tissues [60,61,62,63], as well as in a range of experimental models of SCI (induced by contusion, compression, or by penetration/laceration-type injuries) [26,27,60,64,65,66]. It is possible that reactive perineurial cells may migrate over a framework of locally deposited ECM molecules (e.g., fibronectin, collagen, and fibrin) to support such behaviour [67]. Perineurial cells have been reported to express alpha 3 integrin in vivo as well as alpha 2 and alpha 5 integrins in vitro, which recognise fibronectin, laminin, and various collagen sub-types [68]. Studies have also demonstrated that collagen types I, II, and III (and their degradation products) act as chemotactic signals for migrating fibroblasts [69]. The present demonstration of reactive PNLC that were closely apposed to the inner surface of the dura mater as well as surrounding the implanted scaffold might indicate type-I collagen as a preferred ECM substrate for their migration.

Cords or nests of fibroblast-like cells similar to those described in the present study have also been reported following the transplantation of leptomeningeal cells into experimentally demyelinated areas of adult rat CNS. Ultrastructural characterization of the transplanted cells revealed features such as large rounded or oval nuclei surrounded by a dense cytoplasm containing numerous large Golgi complexes, ribosomal rosettes, and mitochondria, but with small amounts of rough and smooth endoplasmic reticulum. Tight junctions and desmosomes were reported to be abundant between the transplanted cells and processes, and even collagen fibril-containing lacunae were described; however, plasma membrane-associated caveolae and basal lamina were rarely observed [70]. Although the ultrastructural characterization of transplanted leptomeningeal cells is, in some aspects, similar to that of PNLC, the presence of numerous caveolae and the discontinuous or partial basal lamina observed in the scarring cells of the present study appear to be consistent (if subtle) differences between these cell types. Furthermore, our own observations of the fine, closely overlapping processes of reactive meningeal cells located at the inner-most aspect of the damaged meninges revealed the presence of occasional tight junctions, and a cytoplasm containing scattered but consistently dilated rER. There was no basal lamina or the presence of a reformed glia limitans detected on the surface of these cells (likely due to the substantial fluid-filled space that separated the inner meningeal surface from the outer astrocytic surface), and membrane-associated vesicles were rare. Experimental lesions of the CNS that penetrate the meninges have been reported to result in the proliferation and migration of leptomeningeal cells into the injury site, leading to the reformation of the glia limitans at the meningeal cell–astrocyte interface as part of the healing process [3,25,71]. Such injuries have also highlighted similarities between reactive leptomeningeal cells and reactive perineurial cells, this notion being supported by the early observation that the perineurium is regarded as an anatomical continuation of the leptomeninges [72,73], and that their responses to injury reflect a general pattern of fibroblast-like cell activation in both CNS- and PNS-related connective tissues. The distinction between leptomeninges and perineurium has been further blurred by the suggestion that perineurial cells are a form of epitheloid cells that are closely related to arachnoid cells [74], that human meningeomas, meningothelial hyperplasia, and perineuriomas may all be derived from arachnoid cap cells [56,75,76], and that the region of spinal nerve roots where the leptomeninges meet the perineurium are associated with the appearance of so called “transitional arachnoperineurial tissue” [77]. These continuing discussions highlight the difficulties of defining identity and inter-relationships of pial arachnoidal cells, transitional arachnoperineurial cells, and perineurial cells. Nonetheless, the present description of the ultrastructural features of the scarring cells, their locations in and around damaged spinal nerve roots, and their extension along the transition zone strongly suggest they had adopted a perineurial-like phenotype.

Transection injuries of the adult rat brain and dorsal spinal cord have resulted in a marked increase in cells expressing the chemorepellent molecule, SEMA 3, which was associated with reactive leptomeningeal fibroblasts as well as reactive perineurial cells of injured spinal nerve roots [25,78]. An up-regulation of SEMA 3A and SEMA 3F expression was similarly described in epineurial and perineurial fibroblasts at the lesion site and in the distal nerve stump of injured adult rat sciatic nerves [79,80]. These observations were also supported by the identification of increased levels of SEMA 3A being detected in perineurial fibroblasts several months after obstetric branchial plexus trauma-induced neuroma [81]. More recent investigations using genetically engineered mice to study the cellular composition of connective tissue or stromal, non-glial, scarring following experimental SCI and fibrotic scarring after acute brain injury in rat have revealed substantial contributions by type-A pericytes and (PDGFR-ß-positive) perivascular fibroblasts [28,29,82]. Soderblom and colleagues used the alpha1(I) collagen (Col1α1) gene promoter linked to a GFP reporter gene to identify collagen 1α1-expressing cells, and demonstrated that leptomeninges and perivascular fibroblasts contributed to fibroadhesive scarring after partial transection injuries of the adult mouse spinal cord [29]. However, the use the alpha1(I) collagen (Col1α1) gene promoter would also presumably highlight perineurial cells, which have been shown to express this gene [83]. Indeed, the results published by Soderblom and colleagues strongly suggest the presence of intensely GFP-positive perineurium surrounding spinal nerve roots (e.g., their Figure 2A, [29]).

The present data demonstrated clusters or nests of PNLC that were either separated from other structures in the lesioned spinal cord or were found to be partially or completely surrounding small-diameter blood vessels. The ultrastructural features of these cells were identical to those found elsewhere, such as in the reactive perineurium, the interfascicular septae, the transition zone between the implanted collagen scaffold, and the surrounding host spinal cord parenchyma, as well as along the lateral tissue bridge lining the inner aspect of the dura mater. These cells were ultrastructurally dissimilar to those of pericytes that were still closely associated with the microvasculature, or even to pericytes that had become dissociated from the vessel walls (possibly reflecting the migrating/scarring type-A pericyte, as described by Goritz and colleagues [28]). These pericytes demonstrated nuclei with a consistently pale, scattered pattern of euchromatin, and relatively little cell body cytoplasm which was, nonetheless, rich in free ribosomes. The cell bodies and fine overlapping processes of these migrating cells also typically contained expanded rER, which has long been regarded as an indicator of increased protein synthesis and secretion following cell activation [82,84]. It seems reasonable to suggest that the relative contributions of all these cell types (i.e., blood vessel-associated fibroblasts, type-A pericytes, leptomeningeal cells, and PNLC) to fibroadhesive scarring likely depend on the type and severity of SCI, as well as the presence (and most likely type) of implanted bioengineered scaffolds.

Although the present investigation describes a novel aspect of the host scarring reaction to implantation of a type-I collagen scaffold, it is possible that a similar response may also be generated following the use of other (natural or synthetic) polymers. For example, others have also demonstrated layers of DAPI-labelled, GFAP-negative cells that effectively separated reactive astrocytes from an implanted biodegradable polyhydroxybutyrate (PHB) scaffold [85]. Similar GFAP-negative areas separated reactive astrocytes from implanted scaffolds made of chitosan, alginate, or a combination of the two following implantation into experimental rat SCI; however, the authors failed to perform an immunohistochemical stain to identify the type of cells occupying this region [86]. The similarities between the above-mentioned examples of the formation of a narrow transition zone around the implanted scaffold and that demonstrated in the present investigation are striking. It is possible that the detrimental encapsulating PNLC host response described in the present investigation is not limited to the use of collagen-based scaffolds in experimental SCI, but may also extend to other engineered devices that are intended for use following severe traumatic SCI.

## 4. Materials and Methods

### 4.1. Experimental Animals

The surgical procedure and animal handling were performed at the Institute of Neuroscience, Group of Neuropharmacology, Université Catholique de Louvain (UCLouvain), Belgium, according to the EU directive of 22 September 2010 and approved by the local ethical committee on animal experimentation (2014/UCL/MD/012) and by the Belgian authority on animal experimentation (LA1230618). All surgical procedures were performed on adult female Sprague–Dawley rats (*n* = 16, body weight 180–200 g) that were bred in the local UCLouvain animal facility. Experimental animals were housed in standard Makrolon cages (2–3 animals per cage) under 12:12 h light/dark cycles. Food and water were provided ad libitum. All measures were taken to minimise the number of animals used, and to prevent pain and discomfort during the experiments.

### 4.2. Surgical Procedure

Experimental spinal cord resection injuries were performed as described previously [30,32]. Briefly, a subcutaneous injection of buprenorphine (0.1 mg/kg body weight) was given to all animals 30–60 min prior to surgery. Anaesthesia was induced by isoflurane inhalation delivered by a U-400 anaesthesia unit (Agntho’s, Lidingö, Sweden, 4–5% mixture in air for induction and maintained with a 2% mixture). In order to prevent corneal drying, ophthalmic ointment was applied. The shoulder and neck areas were then shaved and disinfected, followed by a mid-line skin incision and blunt dissection of the neck musculature to expose the C3–C4 vertebrae. Using a dissection microscope, a right-sided hemi-laminectomy was performed and a small dural window was opened with micro-scissors, followed by a right-sided, 2 mm long lateral funiculotomy at level C4. Completeness of the resection injury was checked microscopically after aspiration and purging of the resection gap. During surgery, care was taken to prevent severe bleeding and excessive local ischemia by sparing major spinal cord blood vessels. Experimental animals were randomly allocated into two different groups. Control animals (*n* = 8) received the lesion, and having established haemostasis, the dura mater was repaired using 10/0 sutures (Ethicon, Inc., Somerville, MA, USA). The experimental group (*n* = 8) received the extra step of implantation of a sterile, micro-structured hemi-cylinder of type-I collagen scaffold (Optimaix^®^, Matricel GmbH, Herzogenrath, Germany) with its longitudinally orientated pores following the long axis of the spinal cord, before dural repair. The scaffold was provided as individual discs (dimension) housed within sealed 16-well tissue culture plates. Cylinders were prepared using a sterile 2 mm biopsy punch and were hemisected along their long axis with a sterile scalpel to generate scaffolds that were ready for implantation (Figure 1). The layers of neck musculature and skin were realigned and sutured with 6/0 and 4/0 Prolene^®^, respectively (Ethicon Inc., Somerville, MA, USA).

### 4.3. Tissue Processing and Staining, Light Microscopy, and Transmission Electron Microscopy

At 10 weeks p.o., animals were sacrificed in a carbon dioxide euthanasia chamber. Having established respiratory arrest, the vasculature of the animals was cleared of blood by transcardial perfusion with 100 mL phosphate-buffered saline (PBS), followed by 200 mL 4% paraformaldehyde (PFA) in 0.1 M phosphate buffer (for paraffin embedding or cryoprotection/freezing of blocks of spinal cord material, *n* = 3 per group) or 200 mL buffered 3.6% glutaraldehyde (for semi-thin and ultra-thin section, *n* = 5 per group). Tissue blocks approximately 1 cm in length, centred around the lesion/implantation site, were dissected and post-fixed in the same fixative for 24 h (at 4 °C for wax embedding, and at room temperature for TEM). Smaller transverse blocks of lesioned spinal cord (up to 3 blocks per animal, approximately 3–4 mm in length) were then prepared and processed for either wax or Epon embedding.

Transverse (3 µm thick) microtome sections were prepared of the PFA-fixed, paraffin-embedded spinal cord blocks which were then dewaxed, rehydrated, and stained with H&E for general histological observations, as described elsewhere [33]. For electron microscopy, glutaraldehyde-fixed spinal cord blocks were post-fixed with 1% OsO_4_ in 0.1 M phosphate buffer, dehydrated, and embedded in epoxy resin. Semi-thin sections (approximately 1 µm thick) were cut on an ultra-microtome, stained with (0.016 M) toluidine blue (Sigma Aldrich, Taufkirchen, Germany), and coverslipped. For the toluidine blue stains, a minimum of 10 slides per animal (each containing 3 sections) were used for qualitative microscopic examinations. Representative images of toluidine blue- and H&E-stained sections were captured using a Zeiss^®^ Axioplan microscope connected to a Zeiss^®^ AxioVision CCD camera and Zeiss^®^ AxioVision 4.8 software (Carl Zeiss Microscopy GmbH, München, Germany).

For TEM, transverse ultra-thin sections (approximately 150 nm thick) were cut with a Leica Reichert Ultracut S microtome (Leica^®^, Wetzlar, Germany) using a diamond knife (Diatome, Leica^®^, Wetzlar, Germany). Sections were then placed on copper grids and contrasted with 0.5% uranyl acetate (Electron Microscopy Science, Shirley, NY, USA), as described previously [55]. Visualisation by TEM was performed by using a Philips CM10 transmission electron microscope (Philips, Eindhoven, The Netherlands) with an accelerating voltage of 40–100 kV. Representative images were captured using a Morada digital camera.

Immunohistochemistry for GFAP was performed on longitudinal cryostat sections of the spinal cord (20 µm thick), as described earlier [87]. Briefly, a 10 min incubation to block endogenous peroxidase activity (0.3% H_2_O_2_ in PBS containing 0.05% NaN_3_) was followed by washes in PBS (3 × 5 min) and a 1 h serum block in 5% normal goat serum in antibody diluent (PBS containing 1% bovine serum albumin and 0.5% Triton X-100). Sections were then incubated overnight at room temperature with primary rabbit anti-glial fibrillary acidic protein antibody (GFAP, 1:1000, DAKO) in antibody diluent. The next day, sections were washed in PBS, incubated for 1 h in biotinylated goat anti-rabbit secondary antibody (1:500 Vector Laboratories), followed by the Vector ABC technique for peroxidase staining, and visualised with 3,3′-diaminobenzidine.

## 5. Conclusions

The present histological and ultrastructural findings strongly suggest a novel cellular contribution to the scarring response in resection/implantation models of SCI. Reactive PNS-derived, perineurial-like cells from locally damaged spinal nerve roots appeared to extend along the interface between the implanted type-I collagen scaffolds and the surrounding host spinal cord tissue, where they formed a transition zone adjacent to the astrogliotic scar, and effectively encapsulated the scaffold and limited host–implant integration. This type of scarring interface prevents proper implant–host integration, thereby preventing the scaffold’s originally intended bridging role across the lesion site and leading to a permanent functional deficit. The ultrastructural features of these scarring cells are strikingly similar to those of perineurial cells. However, cell location and ultrastructure cannot provide a definitive identification of these cells, hence our preferred use of the term perineurial-like cells. The lesion-only control preparations also revealed the PNLC lining the inner-most aspect of the repaired dura mater. This novel aspect of the cellular composition of damaged, reactive tissue highlights the unexpectedly complex nature of fibroadhesive scarring to traumatic SCI, as well as the implantation of bioengineered collagen scaffolds.

## Data Availability

The data presented in this study are available on request from the corresponding author.

## Supplementary Materials

The following supporting information can be downloaded at: https://www.mdpi.com/article/10.3390/ijms23063221/s1.
