# Supplementary material for: Characterization of a Novel Aspect of Tissue Scarring Following Experimental Spinal Cord Injury and the Implantation of Bioengineered Type-I Collagen Scaffolds in the Adult Rat: Involvement of Perineurial-like Cells?
Source: Int J Mol Sci. 2022 Mar 16;23(6):3221. doi: 10.3390/ijms23063221 (PMC8954100; doi:10.3390/ijms23063221)
Supplement: Supplementary file 1 [file ijms-23-03221-s001.zip › ijms-1588694-supplementary.pdf]

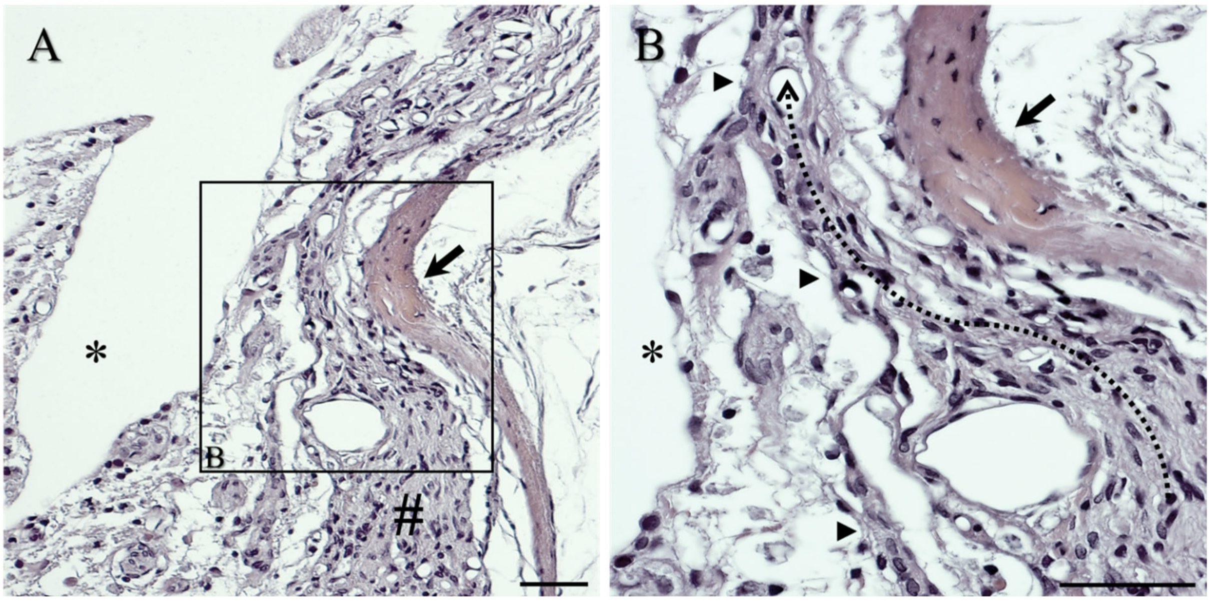

Figure S1. General morphology of lesion sites in control, lesion-only animals, in transverse sections stained for H&E.

(**A**, **B**) In lesion-only animals, multiple layers of reactive cells formed a band of tissue that extended from the edge of the damaged spinal nerve root (indicated by #) and followed a trajectory that followed the inner aspect of the repaired eosinophilic dura mater (arrow) and formed a sheet of cells at the lateral edge of the cystic cavities (asterisk). The trajectory of the migrating reactive cells is indicated by the orientation of ovoid stained nuclei (arrowhead) and the meandering arrow in B**.** Scale bars: A, B = 50 µm.

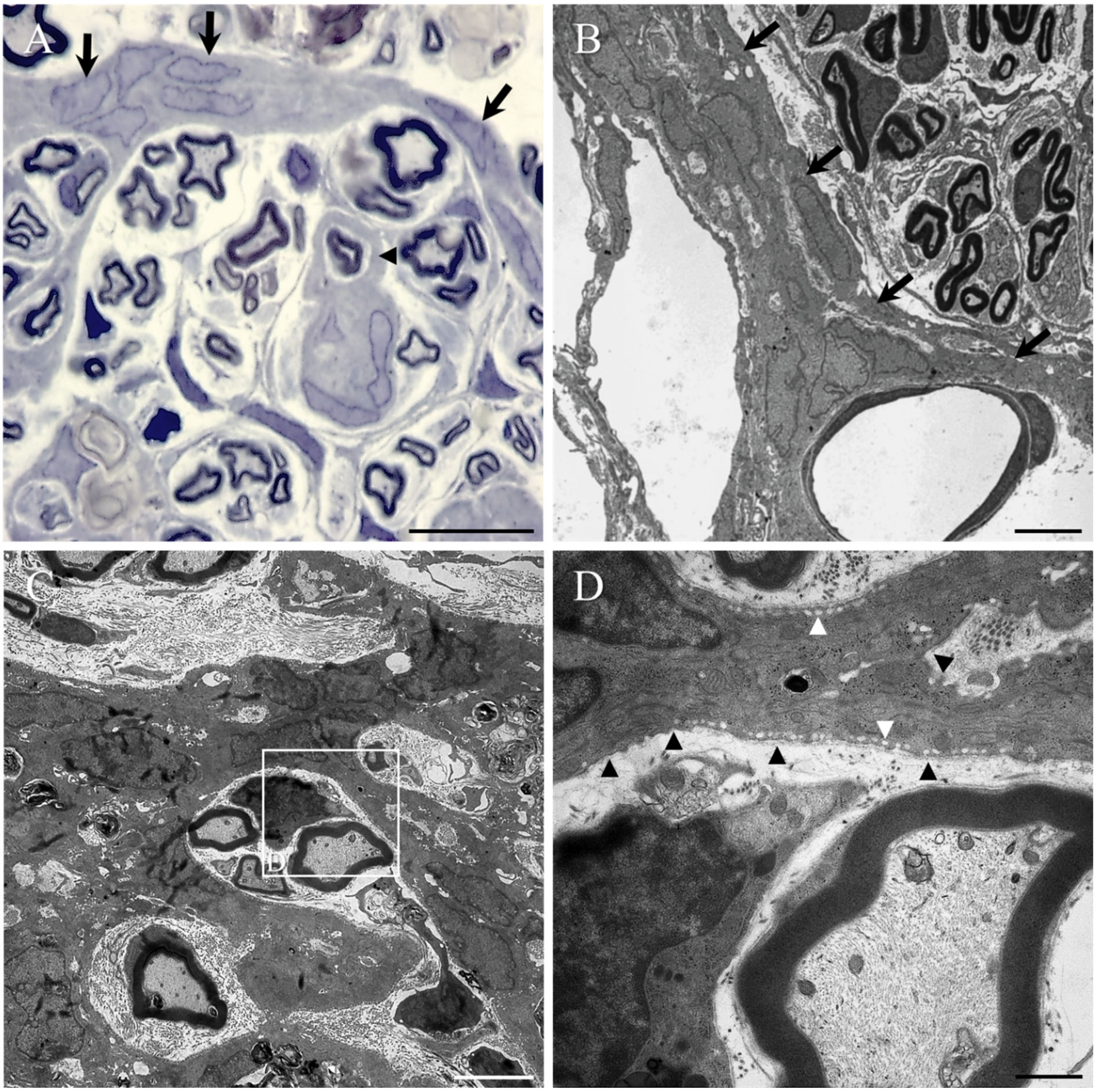

Figure S2. Details of light and electron microscopic changes of the lesioned/regenerated spinal nerve roots. (**A**) Closer inspection of the toluidine-blue stained sections demonstrated that the large, often ovoid nuclei of the thickened, reactive perineurium all displayed a uniform morphology with a medium pale euchromatin surrounded by a thin layer of heterochromatin (black arrows). Cells with the same nuclear morphology and staining intensity as well as their extensive cytoplasm and processes formed the intra-nerve root septa that surrounded regenerating mini-fascicles of Schwann-cell myelinated axons as well as individual myelinated axons (single black arrowhead). Regenerating mini-fascicles were also surrounded by extremely fine processes of fibroblast-like cells that possessed much more darkly stained nuclei and resembled endoneurial fibroblasts. (**B**) Transmission electron microscopy of multiple overlapping cells of the reactive perineurium (arrows). (**C**) Processes of the reactive cells that form the intra-nerve root septae can be seen extending around small mini-fascicles of Schwann-cell myelinated axons or even individual myelinated axons. (**D**) High magnification of boxed area in C. The plasma membrane surfaces of these cells display numerous pinocytic vesicles or caveolae (white arrowheads) and a poorly developed or incomplete basal lamina (black arrowheads). Scale bars: A = 20 µm; B, C = 5 µm; D = 1 µm.

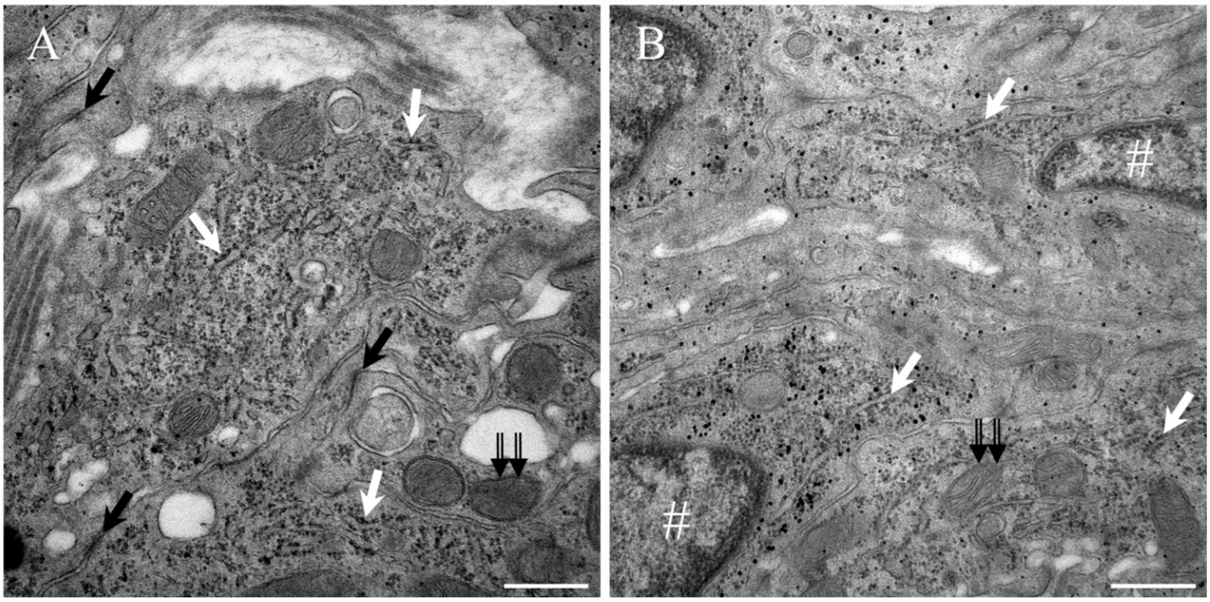

Figure S3. Examples of TEM of PNLC.

(**A**) The presence of abundant free ribosomes, rER (white arrows) of relatively normal dimensions, numerous mitochondria (double black arrow) and tight junctions (e.g. black arrow) were routinely observed within the cytoplasm of PNLC. (**B**) The exposure chosen for this figure differentiates the intensely black glycogen granules from the paler, grey free ribosomes within the cytoplasm of a PNLC cell body. The nucleus (indicated by #), numerous mitochondria (double black arrow) and rER (white arrows) are also demonstrated. Scale bars: A, B = 500 nm.

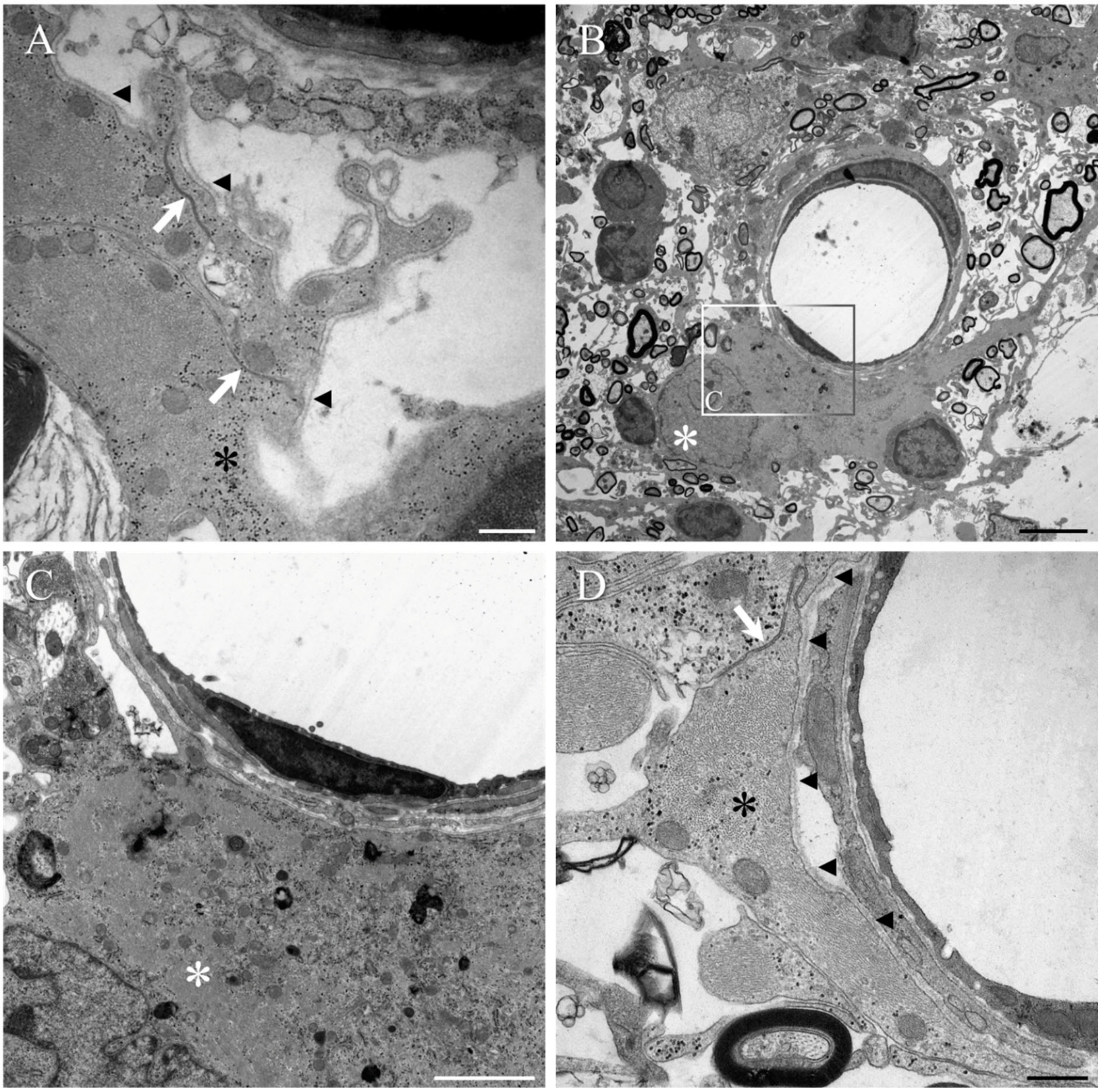

Figure S4. TEM of reactive astrocytes in the areas of damaged spinal cord close to the implanted scaffold. (**A**) Other important local and reactive cell populations in the damaged spinal white matter showed different ultrastructural features. The cytoplasm of reactive hypertrophic astrocytes contained densely packed intermediate filaments and was rich in glycogen granules (asterisk). The plasma membrane was often associated with gap junctions between adjacent astrocytic processes (white arrows) and the free surface was also covered by a well-developed and continuous basal lamina (black arrowheads). (**B**) Reactive astrocyte (white asterisk), in close contact with a capillary within the damaged spinal tissue. (**C**) Higher magnification of the boxed area in B**.** The cell body of the reactive astrocyte is densely packed with intermediate filaments (white asterisk). (**D**) End-foot of a reactive astrocyte (black asterisk) partially surrounding a small venule. The intermediate filament-rich cell cytoplasm contains numerous mitochondria, and the abluminal surface is coated with a thick, well developed basal lamina (arrowheads). A gap junction can be observed between the plasma membrane of the end-foot and an adjacent glycogen-rich cell process (white arrow). Scale bars: A, D = 500 nm; B = 5 µm; C = 2 µm.

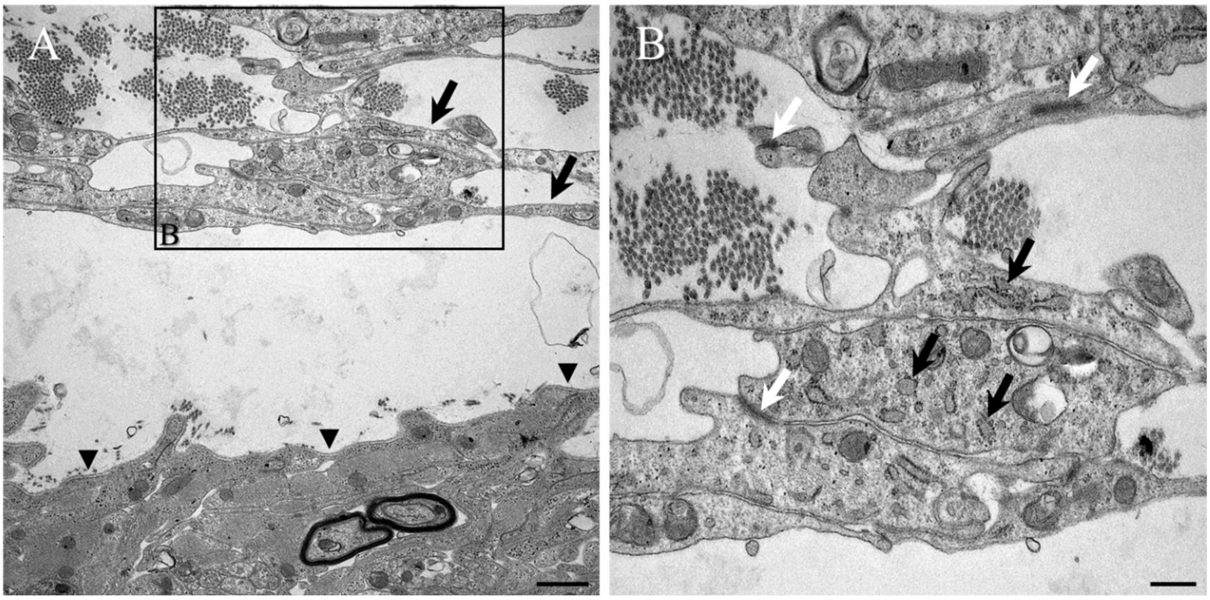

Figure S5. TEM of the reactive leptomeninges.

(**A**) Demonstration of the ultrastructural features of reactive leptomeningeal cells following spinal cord injury. The inner most layers of the reactive leptomeninges were composed of loosely overlapping electron lucent process (black arrows) with large and small lacunae containing bundles of collagen fibrils. The layers of reactive leptomeninges were separated from the surface of the nearby and basal lamina-coated glia limitans (black arrowheads) by a fluid-filled gap. (**B**) High magnification of boxed area in A. Adjacent electron lucent process of the reactive leptomeninges were often connected by tight junctions (white arrows) and contained numerous mitochondria and scattered intermediate filaments. The cytoplasm of these reactive process was striking in its abundance of rER with widened cisterna (back arrows), showing strong similarities to the morphology of reactive pericytes. Scale bars: A = 1 µm; B = 500 nm.
